# EGF-like growth factors upregulate pentraxin 3 expression in human granulosa-lutein cells

**DOI:** 10.1186/s13048-024-01404-5

**Published:** 2024-05-08

**Authors:** Yuxi Li, Hsun-Ming Chang, Hua Zhu, Ying-Pu Sun, Peter C. K. Leung

**Affiliations:** 1 Department of Obstetrics and Gynecology, BC Children’s Hospital Research Institute, University of British Columbia, Room 317, 950 West 28th Avenue, Vancouver, BC V5Z 4H4 Canada; 2 Center for Reproductive Medicine, The First Affiliated Hospital of Zhengzhou University, 40, Daxue Road, Zhengzhou, 450052 Henan China; 3 Henan Key Laboratory of Reproduction and Genetics, The First Affiliated Hospital of Zhengzhou University, Zhengzhou, China; 4 Henan Provincial Obstetrical and Gynecological Diseases (Reproductive Medicine) Clinical Research Center, The First Affiliated Hospital of Zhengzhou University, Zhengzhou, China; 5 Reproductive Medicine Center, Department of Obstetrics and Gynecology, China Medical University Hospital, Taichung, Taiwan

**Keywords:** Amphiregulin, Betacellulin, Epiregulin, Pentraxin 3, ERK1/2 signaling, Human granulosa cell

## Abstract

The epidermal growth factor (EGF)-like factors, comprising amphiregulin (AREG), betacellulin (BTC), and epiregulin (EREG), play a critical role in regulating the ovulatory process. Pentraxin 3 (PTX3), an essential ovulatory protein, is necessary for maintaining extracellular matrix (ECM) stability during cumulus expansion. The aim of this study was to investigate the impact of EGF-like factors, AREG, BTC, and EREG on the expression and production of PTX3 in human granulosa-lutein (hGL) cells and the molecular mechanisms involved. Our results demonstrated that AREG, BTC, and EREG could regulate follicular function by upregulating the expression and increasing the production of PTX3 in both primary (obtained from 20 consenting patients undergoing IVF treatment) and immortalized hGL cells. The upregulation of PTX3 expression was primarily facilitated by the activation of the extracellular signal-regulated kinase 1 and 2 (ERK1/2) signaling pathway, induced by these EGF-like factors. In addition, we found that the upregulation of PTX3 expression triggered by the EGF-like factors was completely reversed by either pretreatment with the epidermal growth factor receptor (EGFR) inhibitor, AG1478, or knockdown of EGFR, suggesting that EGFR is crucial for activating the ERK1/2 signaling pathway in hGL cells. Overall, our findings indicate that AREG, BTC, and EREG may modulate human cumulus expansion during the periovulatory stage through the upregulation of PTX3.

**Supplementary Information:**

The online version contains supplementary material available at 10.1186/s13048-024-01404-5.

## Introduction

The cumulus-oocyte-complex (COC) matrix consists of human oocytes surrounded by cross-linked hyaluronan (HA) extracellular matrix with cumulus cells. Proper assembly, stability, and physical properties of this matrix are crucial for successful ovulation. The interaction between HA and HA-organizing factors plays a critical role in the transport of the COC to the oviduct and fertilization [1]. Three key factors, Inter-inhibitor [2], the secreted product of tumor necrosis factor-stimulated gene-6 (TSG-6) [3], and pentraxin 3 (PTX3) [4] have been identified as essential for the correct assembly of the COC matrix due to their interactions with HA and each other.

Pentraxin 3 (PTX3) belongs to the long pentraxin superfamily and forms an octameric complex as a glycoprotein [5]. Studies have shown that PTX3 expression is associated with oocyte quality and embryo development potential obtained from women with polycystic ovary syndrome [6]. After the LH surge, PTX3 expression is specifically upregulated by cumulus granulosa cells surrounding the oocyte, playing critical roles in cumulus cell-oocyte (COC) interactions during the periovulatory period. In mice lacking PTX3 (*Ptx3*-/-), cumulus cells fail to establish a functional matrix, but this can be rescued by adding recombinant PTX3 ex vivo [4]. Although PTX3 does not directly bind to hyaluronan (HA), it plays an essential role in assembling the HA polymer in the cumulus oophorus extracellular matrix (ECM) by binding with TSG-6 [1] or the heavy chains of inter-α-trypsin inhibitor (IαI) [7], forming multimolecular complexes that cross-link HA chains. These findings highlight the significance of PTX3 as an HA-organizing factor in human cumulus maturation and expansion during ovulation.

The epidermal growth factor (EGF) family, which includes amphiregulin (AREG), epiregulin (EREG), betacellulin (BTC), EGF, heparin-binding EGF-like growth factor (HB-EGF), epigen (EPGN), transforming growth factor-α (TGFα), and the neuregulins 1–4 (NRG1-4), consists of members with highly similar structures and functions [8]. Among these members of the EGF-like growth factor, the mRNA levels of AREG, BTC, and EREG are initially undetectable in the ovary before the LH surge. However, following LH activation, they are specifically upregulated, returning to basal levels thereafter [9]. In response to the LH signal, AREG, BTC, and EREG induce extensive gene activations that play essential roles in oocyte meiotic maturation, cumulus expansion, and ovulation [10]. Our previous studies have demonstrated that BTC promptly induces the phosphorylation of connexin 43 at Ser368, resulting in decreased gap junctional intercellular communication (GJIC) activity in human granulosa-lutein (hGL) cells and regulating oocyte meiotic resumption [11]. Additionally, the addition of AREG, BTC, and EREG to rat follicle cultures increases the expression of genes related to cumulus matrix formation (*COX-2*, *HAS-2*, and *TSG-6*), promoting cumulus expansion [12].

EGF-like peptides transmit LH signals mainly by activating the epidermal growth factor receptor (EGFR), also known as HER1 or ErbB1. EGFR, a transmembrane receptor tyrosine kinase (RTK) with a ligand-binding extracellular domain, a single membrane-spanning region, and an intracellular tyrosine, belongs to the ErbB family with four members [13]. Blocking EGFR activity disrupts oocyte meiotic resumption, cumulus expansion, and ovulation, emphasizing the significant role of EGFR in LH signaling [14]. EGF family members activate EGFR through several signaling pathways, including MAP kinase (MAPK), phosphatidylinositol-3 kinase (PI3K), and Janus kinase/signal transducers and activators of transcription (JAK/STAT) pathways. A critical downstream factor in EGFR-mediated EGF family ligand signaling is the extracellular signal-regulated kinase 1 and 2 (ERK1/2), also known as MAPK3/1. Activation of EGFR-ERK1/2 by AREG, BTC, and EREG is essential in the ovulatory process, as it promotes meiotic resumption, ovulation, cumulus expansion, oocyte mRNA translation, and luteinization [11, 12, 15].

While the roles of gonadotropins, cytokines, and oocyte-specific growth factors in regulating PTX3 expression have been studied, the modulation of PTX3 expression by granulosa cell-derived EGF-like growth factors remains undefined. Considering the essential functions of EGF-like factors in various ovulatory processes and the importance of PTX3 in cumulus expansion, we aim to investigate the effects of AREG, BTC, and EREG on PTX3 expression and production using human granulosa-lutein (hGL) cells and explore the potential underlying mechanisms of these effects.

## Materials and methods

### Antibodies and reagents

Monoclonal mouse anti-Phospho-ERK1/2 (Thr202/Tyr204) (^#^9106) (1:1000) and polyclonal rabbit anti-ERK1/2 (^#^9102) (1:1000) antibodies were obtained from Cell Signaling Technology (Beverly, MA). Monoclonal mouse anti-α-tubulin (B-5-1-2) (sc-23,948) (1:3000) antibodies were obtained from Santa Cruz Biotechnology (Santa Cruz, CA). Horseradish peroxidase-conjugated goat anti-rabbit IgG and goat anti-mouse IgG were obtained from Bio-Rad Laboratories (Hercules, CA). Recombinant human AREG, BTC, and EREG were obtained from R&D Systems (Minneapolis, MN). AG 1478 was obtained from Sigma-Aldrich Corp. U0126 was obtained from Calbiochem (San Diego, CA).

### Culture of Simian virus 40 large T antigen–immortalized human granulosa (SVOG) cells

In this study, we utilized the Simian virus 40 large T antigen–immortalized human granulosa (SVOG) cell line, which is a non-tumorigenic immortalized human granulosa cell line. The SVOG cell line was created by transfecting primary hGL cells obtained from females undergoing in vitro fertilization (IVF) with the SV40 large T antigen [16]. Previous studies have demonstrated that SVOG cells exhibit similar characteristics and responses to primary hGL cells [16, 17]. Thus, for the current investigation, we employed SVOG cells to assess the effects of EGF-like factor treatment. To initiate the experiments, cells were counted using a hemocytometer, and cell viability was determined using 0.04% trypan blue staining. Trypan blue serves as a cell-impermeant stain utilized for assessing the quantity of deceased cells within a viable cell population. Its effectiveness lies in its property as a charged dye, which prevents its penetration into intact cell membranes. Living cells repel the dye, whereas non-viable cells or those with compromised membranes absorb it, resulting in a distinctive intense blue staining. The SVOG cells were then seeded at a density of 5 × 10^5^ cells per well in 6-well plates and cultured in a humidified atmosphere containing 5% CO2 and 95% air at 37 °C. The culture medium used was Dulbecco’s Modified Eagle’s Medium/nutrient mixture F-12 Ham (DMEM/F-12; Sigma-Aldrich Corp., Oakville, ON) supplemented with 10% charcoal/dextran-treated fetal bovine serum (HyClone, Logan, UT), 100 µg/ml streptomycin sulfate (Invitrogen, Life Technologies), 100 U/ml penicillin (Invitrogen, Life Technologies, NY), and GlutaMAX (1X, Invitrogen, Life Technologies). The medium for SVOG cells was refreshed every other day to maintain their viability and growth. The confluence of the cells before being utilized in experiments consistently exceeds 95%. The cells were starved by incubating them in serum-free DMEM/F-12 medium for 12 h before the treatment of AREG, BTC or EREG. This ensured that the cells were in a consistent and controlled state before the administration of EGF-like growth factors treatment. In this study, we investigated the effect of EGF-like growth factors on the expression of PTX3. To do this, we treated SVOG cells with 50 ng/ml of AREG, BTC, and EREG separately for different time points (3, 6, or 9 h).

### Preparation and culture of primary granulosa-lutein (hGL) cells

In this study, primary hGL cells were obtained with informed consent from patients following approval from the University of British Columbia Ethics Board. Follicular samples were anonymized promptly post-collection, and each primary culture consisted of cells sourced from a single patient, totaling 20 patients in the study cohort. Two controlled ovarian stimulation protocols were employed for in vitro fertilization patients: (1) luteal-phase nafarelin acetate (Synarel, Pfizer, Kirkland, Quebec, Canada) and (2) follicular phase GnRH antagonist (Ganirelix; Merck Canada) downregulation. Gonadotropin stimulation commenced on menstrual cycle day 2 using human menopausal gonadotropin (hMG; Menopur, Ferring, Canada) and recombinant FSH (Puregon, Merck, Canada). Human chorionic gonadotropin was administrated 34–36 h before oocyte retrieval, based on follicle size. Granulosa cells were purified by density centrifugation from follicular aspirates obtained from women undergoing oocyte retrieval, as previously described [18]. Each sample of primary hGL cells collected from individual females was cultured separately. The purified hGL cells were seeded at a density of 5 × 10^5^ cells per well in 6-well plates with the same culture environment and medium used for the SVOG cell line.

### Reverse transcription and real-time quantitative PCR (RT-qPCR)

Cells were washed with cold phosphate buffered saline (PBS), and total RNA was extracted with TRIzol Reagent (Invitrogen) according to the manufacturer’s instructions. The extracted RNA (2 µg) was reverse-transcribed into first-strand cDNA with random primers and MMLV reverse transcriptase (Promega, Madison, WI). For quantitative real-time polymerase chain reaction (qPCR), each 20-µl reaction contained 1X SYBR Green PCR Master Mix (Applied Biosystems, Foster City, CA), 20 ng cDNA, and 250 nM of each specific primer. The primers used were *pentraxin 3* (*PTX3*), 5′-TCT CTG GTC TGC AGT GTT GG-3′ (sense) and 5′-TGA AGA GCT TGT CCC ATT CC-3′ (antisense); *glyceraldehyde-3-phosphate dehydrogenase* (*GAPDH*), 5’- ATG GAA ATC CCA TCA CCA TCT T -3’ (sense) and 5’- CGC CCC ACT TGA TTT TGG − 3’ (antisense); and *epidermal growth factor receptor* (*EGFR*), 5’-GGT GCA GGA GAG GAG AAC TGC − 3′ (sense) and 5’- GGT GGC ACC AAA GCT GTA TT -3’ (antisense). qPCR was performed on an Applied Biosystems 7300 Real-Time PCR System equipped with a 96-well optical reaction plate (Applied Biosystems). The specificity of each assay was validated by dissociation curve analysis and agarose gel electrophoresis of the PCR products. Assay performance was validated by evaluating the amplification efficiencies through calibration curves and ensuring that the plot of the log input amount vs. ΔCq (also known as ΔCT) had a slope < |0.1|. The PCR parameters were 50 °C for 2 min, 95 °C for 10 min, and 40 cycles of 95 °C for 15 s and 60 °C for 1 min. Three separate experiments were conducted on different cultures, and each sample was assayed in triplicate. The mRNA levels were determined using the comparative Cq (2^–ΔΔCq^) method, with *GAPDH* as the reference gene. The mean value was used for the analysis.

### Western blot analysis

After the treatment, cells were washed with cold PBS 3 times and lysed in lysis buffer (Cell Signaling) containing a protease inhibitor cocktail (Sigma-Aldrich). The lysates were then centrifuged at 20,000 x *g* for 10 min at 4 °C to remove cellular debris. Protein concentrations were quantified using a DC Protein Assay (Bio-Rad Laboratories). Equal amounts of protein were separated by 10% SDS-PAGE and transferred from the gels to polyvinylidene fluoride membranes. Next, the membranes were blocked with 5% nonfat dried milk in a Tris-buffered solution containing 0.05% Tween 20 at room temperature for 1 h. The relevant primary antibodies were then incubated with the membranes at 4 °C overnight. After washing with TBS, the membranes were incubated with a peroxidase-conjugated secondary antibody (Bio-Rad) at room temperature for 1 h. For the detection of immunoreactive bands, either an enhanced chemiluminescence substrate or a SuperSignal West Femto Chemiluminescence Substrate (Pierce, Rockford, IL) was used. The membranes were exposed to CL-XPosure film (Thermo Fisher, Waltham, MA) for visualization of the detected bands. To re-probe the membranes, stripping buffer (50 mM Tris-HCl pH 7.6, 10 mmol/l β-mercaptoethanol, and 1% SDS) was used, and the membranes were incubated at 50 °C for 30 min. Subsequently, the membranes were probed again with a rabbit anti-ERK1/2 antibody, which served as a loading control for normalization.

### Small interfering RNA (siRNA) transfection

The cells were pre-cultured until they reached 50% confluence in antibiotic-free DMEM/F12 medium supplemented with 10% charcoal/dextran-treated fetal bovine serum. Subsequently, they were transfected with either 25 nM EGFR-targeting siRNA (ON-TARGET*plus* SMARTpool) or 25 nM control siRNA (ON-TARGET*plus* Non-targeting Pool) (Dharmacon) using Lipofectamine RNAiMAX (Invitrogen) for a duration of 48 h. The effectiveness of knockdown for each target was confirmed by performing a Western blot analysis.

### Measurement of PTX3

After 9 h of separate treatment with AREG, BTC, or EREG, the cell culture mediums were collected for an enzyme-linked immunosorbent assay (ELISA). The measurement of PTX3 protein production in the culture medium was conducted using quantitative sandwich enzyme immunoassay Quantikine kits (R&D Systems), following the manufacturer’s instructions. To ensure accuracy, the PTX3 levels were normalized to the protein concentration of each cell lysate. The normalized PTX3 levels for each treated sample were then expressed as percentages of the normalized control levels.

### Statistical analysis

For primary cell culture, at least three isolations (*n* ≥ 3) of cells were obtained to perform each in vitro experiment. For the immortalized cell line, we seeded the cells at least 3 separate times (3 different cell passages) and treated them accordingly at least 3 separate times. The numbers of independent experiment were presented in the figure legends. For statistical analysis, the results were subjected to one-way analysis of variance (ANOVA) followed by Tukey’s multiple comparison test in PRISM software from GraphPad Software, Inc. (San Diego, CA). The data are presented as the mean ± SEM of at least three independent experiments. Differences were considered statistically significant if the *P*-value was less than 0.05 (*P* < 0.05). 

## Results

### EGF-like factors upregulate the expression and production of PTX3 in hGL cells

**Fig. 1:**
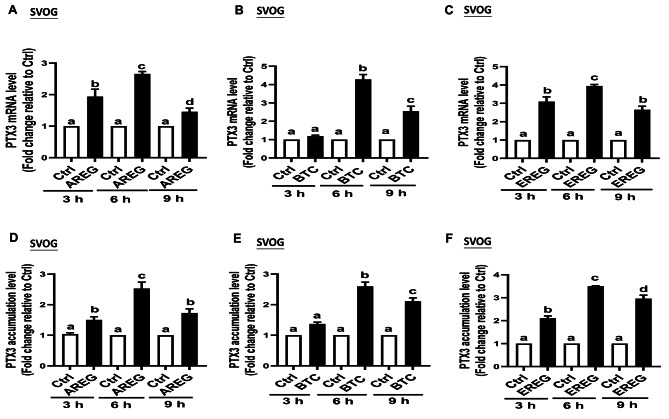
EGF-like factors upregulate the expression and accumulation of PTX3 in SOVG cells. (**A**, **B**, and **C**) SVOG cells were treated with either vehicle control or 50 ng/ml AREG (**A**), BTC (**B**), or EREG (**C**) for different time points (3, 6, or 9 h), and the PTX3 mRNA levels were examined using RT-qPCR (*n* = 4). (**D**, **E**, and **F**) SVOG cells were treated with either vehicle control or 50 ng/ml AREG (**D**), BTC (**E**), or EREG (**F**) for different time points (3, 6, or 9 h), and the PTX3 accumulation levels in the conditioned medium were measured using enzyme immunoassay (*n* = 4). The data presented are the mean ± SEM of at least three independent experiments. Values labeled with different letters are significantly different (*P* < 0.05). AREG, amphiregulin; BTC, betacellulin; EREG, epiregulin; Ctrl, control

**Fig. 2:**
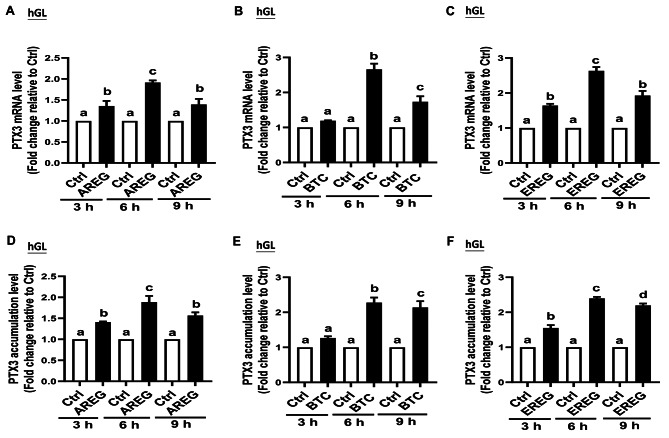
EGFR mediates the EGF-like factors-induced upregulation of PTX3 in primary hGL cells. (**A**, **B**, and **C**) Primary hGL cells were treated with either vehicle control or 50 ng/ml AREG (**A**), BTC (**B**), or EREG (**C**) for different time points (3, 6, or 9 h), and the PTX3 mRNA levels were examined using RT-qPCR (*n* = 4). (**D**, **E**, and **F**) Primary hGL cells were treated with either vehicle control or 50 ng/ml AREG (**D**), BTC (**E**), or EREG (**F**) for different time points (3, 6, or 9 h), and the PTX3 accumulation levels in the conditioned medium were measured using an enzyme immunoassay (*n* = 5). The data presented are the mean ± SEM of at least three independent experiments. Values labeled with different letters are significantly different (*P* < 0.05). AREG, amphiregulin; BTC, betacellulin; EREG, epiregulin; Ctrl, control

The results obtained from reverse transcription-quantitative polymerase chain reaction (RT-qPCR) (Fig. 1A-C) showed a temporal pattern PTX3 mRNA expression following treatment with AREG, BTC, and EREG in SVOG cells. While AREG and EREG follow a similar pattern, the expression pattern differs for BTC. Specifically, PTX3 mRNA levels began to rise at 3 h post-treatment, peaked at 6 h, and subsequently declined by 9 h of culture after treatment with AREG or EREG. Notably, at the 3-hour time point, PTX3 mRNA levels remained relatively unchanged after BTC treatments, whereas a significant increase was observed at 6 h, persisting thereafter in SVOG cells. To further validate the regulatory role of AREG, BTC, and EREG on PTX3, we conducted similar experiments using primary hGL cells obtained from women undergoing IVF. The results from RT-qPCR (Fig. 2A-C) in primary hGL cells mirrored those from the SVOG cells, further confirming the impact of AREG, BTC, and EREG on PTX3 expression.

Next, to determine whether the increase in PTX3 mRNA induced by the EGF-like growth factors corresponded to an increase in PTX3 protein production, we measured the accumulation of PTX3 in the conditioned medium after culturing SVOG cells and primary hGL cells with AREG, BTC, and EREG using enzyme-linked immunosorbent assay (ELISA). We treated the cells with 50 ng/ml of AREG, BTC, and EREG for different time points (3, 6, or 9 h). The results from ELISA (Fig. 1D-F for SVOG cells and Fig. 2D-F for primary hGL cells) corroborated the findings from RT-qPCR. The changes in PTX3 protein levels in the conditioned medium were consistent with the alterations observed in PTX3 mRNA expression in both SVOG cells and primary hGL cells. Overall, these results demonstrate that AREG, BTC, and EREG can regulate the expression of PTX3 at both the mRNA and protein levels in SVOG cells and primary hGL cells, suggesting their significant role in modulating PTX3 expression during the investigated time points. While we conducted experiments and analyses using all three reagents (AREG, BTC, and EREG), we have chosen to primarily emphasize the results derived from BTC treatment in our study. This decision stems from the observation that the treatment of AREG, BTC, and EREG exhibited similar patterns in our experimental outcomes. However, the effects observed following BTC treatment were the most statistically significant among the three reagents. The results of AREG and EREG are provided in Supplemental figures.

### EGFR mediates the upregulation of PTX3 expression induced by BTC in hGL cells

**Fig. 3:**
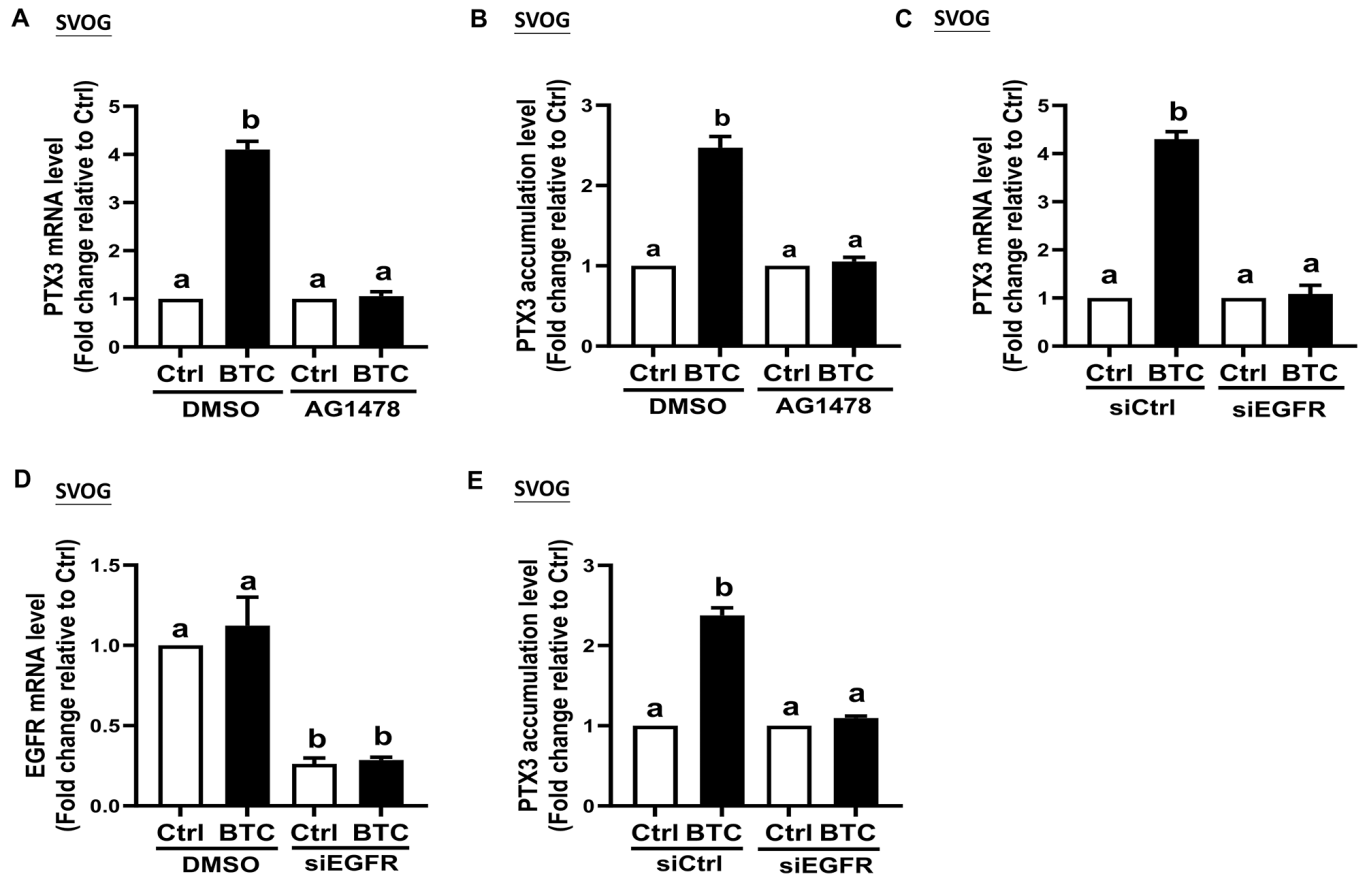
EGFR mediates the BTC-induced up-regulation of PTX3 in SVOG cells. SVOG cells were pretreated with either dimethylsulfoxide (DMSO) or the specific EGFR inhibitor, AG1478 (10 µM) for 1 h and then treated with either a vehicle control or 50 ng/mL of BTC for an additional 6 h. (**A**) The PTX3 mRNA levels were examined using RT-qPCR (*n* = 3). (**B**) The PTX3 accumulation levels in the conditioned medium were measured using enzyme immunoassay (*n* = 4). SVOG cells were transfected with either siCtrl or siEGFR for 48 h before the treatment with BTC (for an additional 6 h). (**C**)€ Changes in PTX3 mRNA levels were examined using RT-qPCR (*n* = 3). (**D**) The knockdown efficiency and specificity of siEGFR were detected using RT-qPCR (*n* = 4). (**E**) The PTX3 accumulation levels in the conditioned medium were measured using an enzyme immunoassay (*n* = 4). The data presented are the mean ± SEM of at least three independent experiments. Values labeled with different letters are significantly different (*P* < 0.05). BTC, betacellulin; Ctrl, control

**Fig. 4:**
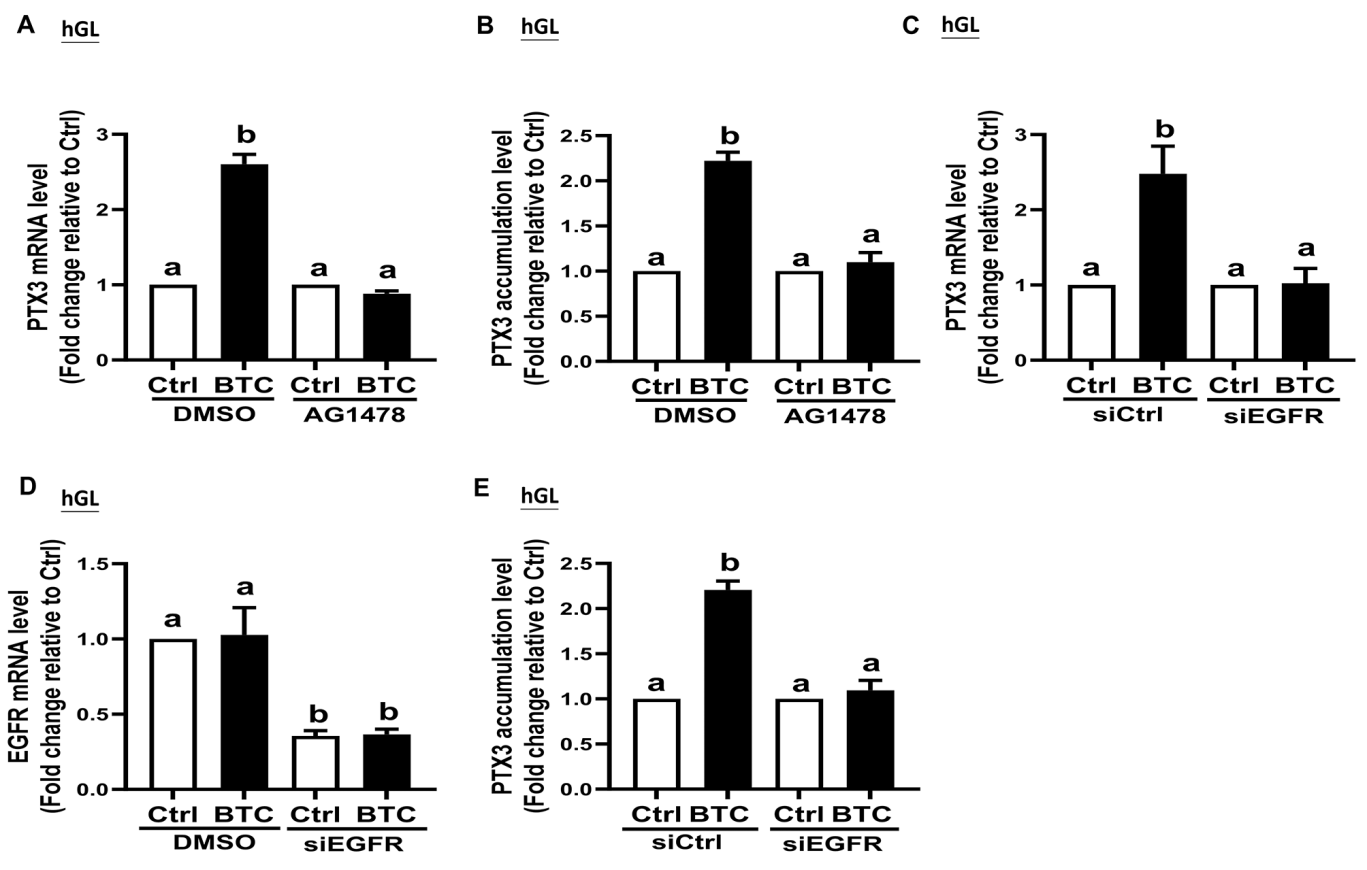
EGFR mediates the BTC-induced up-regulation of PTX3 in primary hGL cells Primary hGL cells were pretreated with either dimethyl sulfoxide (DMSO) or the specific EGFR inhibitor, AG1478 (10 µM) for 1 h and then treated with either a vehicle control or 50 ng/mL of BTC for an additional 6 h. (**A**) The PTX3 mRNA levels were examined using RT-qPCR (*n* = 5). (**B**) The PTX3 accumulation levels in the conditioned medium were measured using an enzyme immunoassay. Primary hGL cells were transfected with either siCtrl or siEGFR for 48 h before the treatment with BTC (for an additional 6 h) (*n* = 4). (**C**) Changes in PTX3 mRNA levels were measured using RT-qPCR. (**D**) The knockdown efficiency and specificity of siEGFR were examined using RT-qPCR (*n* = 4). (**E**) The PTX3 accumulation levels in the conditioned medium were measured using an enzyme immunoassay (*n* = 5). The data presented are the mean ± SEM of at least three independent experiments. Values labeled with different letters are significantly different (*P* < 0.05). BTC, betacellulin; Ctrl, control

To explore the involvement of EGFR, a pharmacological inhibition approach was utilized. The SVOG cells were pretreated with 10 µM AG1478, a specific inhibitor of EGFR, for 1 h before exposure to BTC (50 ng/ml) for different time points (Fig. 3A) and in primary hGL cells (Fig. 4A). The results showed that pretreatment with AG1478 completely abolished the increase in PTX3 mRNA expression induced by BTC in both SVOG cells and primary hGL cells. Similarly, we used a specific siRNA targeting EGFR (siEGFR) to further investigate the role of EGFR in the upregulation of PTX3 induced by AREG, BTC, and EREG in hGL cells. The knockdown of EGFR using siRNA was found to completely abolish the BTC-induced increase in PTX3 mRNA expression in both SVOG cells (Fig. 3C-D) and primary hGL cells (Fig. 4C-D). Furthermore, pretreated with 10 µM AG1478 or siEGFR also blocked the upregulation of PTX3 induced by both AREG and EREG in both SVOG cells (Supplemental Fig. 1A-B and 2 A-D) and primary hGL cells (Supplemental Fig. 1C-D and 3A-D).

To validate these findings, ELISA was performed to measure the PTX3 production in the conditioned medium after treating the cells with BTC. The results from both SVOG cells and primary hGL cells confirmed that the BTC-induced upregulation of PTX3 production was inhibited by either pretreatment with AG1478 (Figs. 3B and 4B) or knockdown of EGFR (Figs. 3E and 4E). These results further supported the hypothesis that EGFR is essential for the upregulation of PTX3 in response to EGF-like factors in hGL cells.

Overall, the study provided evidence that EGFR plays a critical role in mediating the upregulation of PTX3 induced by AREG, BTC, and EREG in hGL cells. These findings highlight the significance of EGFR signaling in the regulation of PTX3 expression and its potential importance in ovulatory processes, including meiotic resumption, ovulation, and cumulus expansion.

### EGFR is responsible for the BTC-induced activation of the MEK/ERK signaling pathway in hGL cells

**Fig. 5:**
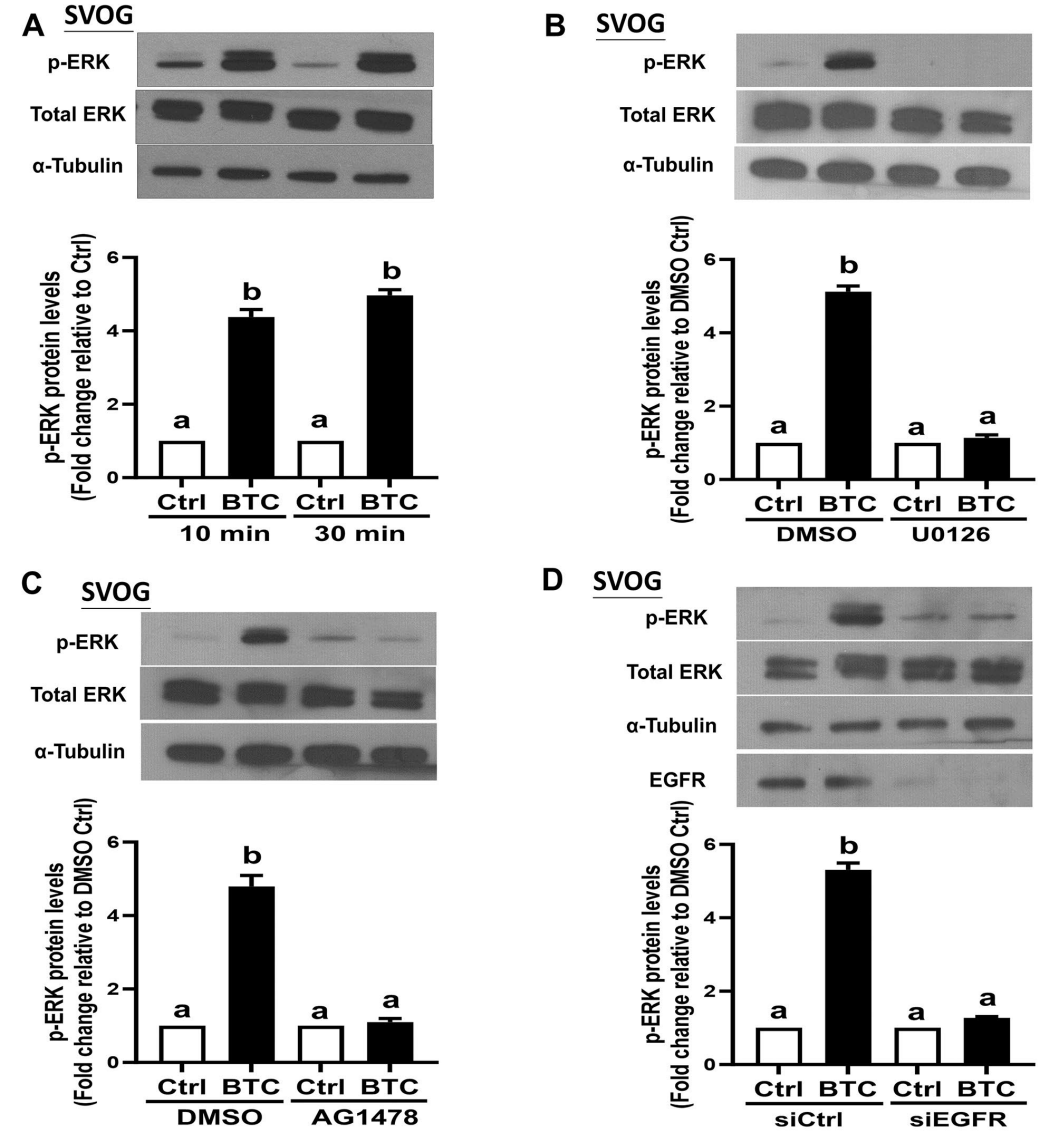
EGFR is responsible for the BTC-induced activation of the MEK/ERK signaling pathway in SVOG cells. (**A**) SVOG cells were treated with either vehicle control or 50 ng/mL BTC for 10–30 min, and the phosphorylated protein levels of ERK1/2 were examined using Western blot analysis (*n* = 4). (**B**) SVOG cells were pretreated with either DMSO or the MEK inhibitor, U0126 (10 µM) for 1 h before the treatment with either vehicle control or 50 ng/mL BTC for an additional 30 min (*n* = 4). (**C**) SVOG cells were pretreated with either DMSO or the EGFR inhibitor, AG1478 (10 µM) for 1 h before the treatment with either a vehicle control or 50 ng/mL BTC for an additional 30 min (*n* = 3). (**D**) SVOG cells were transfected with either siCtrl or siEGFR for 48 h before the treatment of BTC (for an additional 6 h). The phosphorylated protein levels of ERK1/2 were examined via Western blot analysis (*n* = 4). The data presented are the mean ± SEM of at least three independent experiments. Values labeled with different letters are significantly different (*P* < 0.05). BTC, betacellulin; Ctrl, control

Initially, SVOG cells were treated with 50 ng/ml of BTC for different time points (10 min and 30 min). Western blot analysis revealed that the phosphorylation of ERK1/2, a well-known downstream mediator activated by EGF-like growth factors, was evident at both 10 min and 30 min after BTC treatment (Fig. 5A). To further examine the involvement of the MEK/ERK signaling pathway in this process, we used a MEK inhibitor, U0126, as a pretreatment before BTC exposure. The results demonstrated that U0126 completely inhibited the BTC-induced activation of ERK1/2 (Fig. 5B). Similar results were observed when cells were treated with AREG and EREG, which also activated the phosphorylation of ERK1/2, and this effect was abolished by U0126 (Supplemental Fig. 4A-D).

As the study had already established that EGFR mediates the upregulation of PTX3 induced by BTC in hGL cells, we would like to explore whether EGFR is responsible for the activation of the MEK/ERK signaling pathway induced by BTC. To investigate this, we used a specific EGFR inhibitor, AG1478, and a siEGFR-based inhibition approach (siEGFR) to inhibit or knock down EGFR. The results from Western blot analysis showed that either pretreatment with AG1478 (Fig. 5C) or knockdown of EGFR using siEGFR (Fig. 5D) significantly attenuated the BTC-induced phosphorylation of ERK1/2 in SVOG cells. Similarly, treatment with AREG or EREG in SVOG cells yielded comparable effects on the activation of ERK1/2, which were also inhibited by either pretreatment with AG1478 or knockdown of EGFR (Supplemental Fig. 5A-D).

Taken together, these findings suggest that EGFR is responsible for the activation of the MEK/ERK signaling pathway induced by BTC, AREG, and EREG in hGL cells. The activation of this signaling pathway appears to be crucial in mediating the upregulation of PTX3 induced by EGF-like growth factors in these cells.

### BTC-induced MEK/ERK signaling pathway mediates the upregulation of PTX3 in hGL cells

**Fig. 6:**
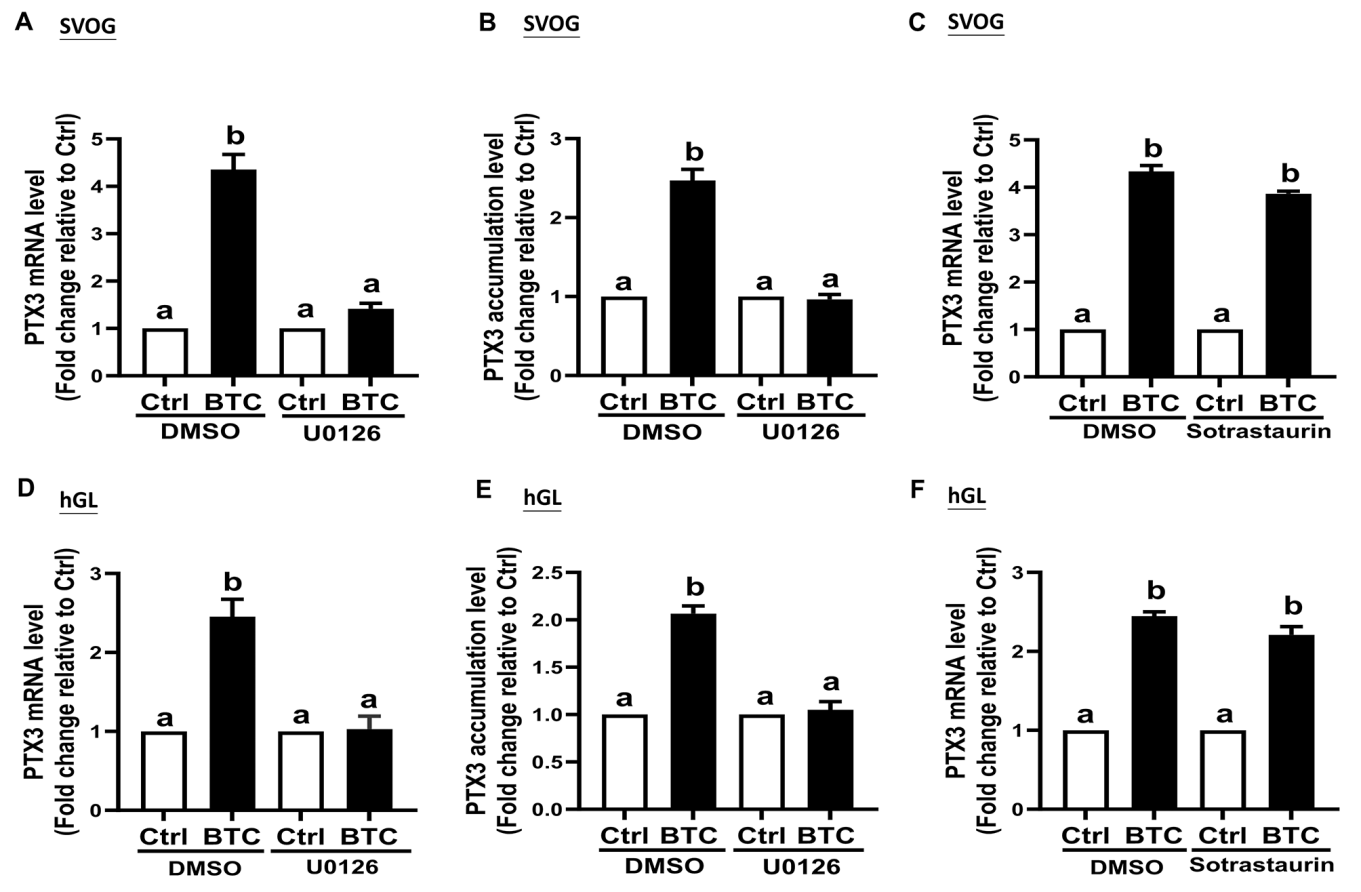
BTC-induced MEK/ERK signaling pathway mediates the upregulation of PTX3 in both SVOG and primary hGL cells. (**A**, **B**) SVOG cells were pretreated with either DMSO or the MEK inhibitor, U0126 (10 µM) for 1 h before the treatment with either vehicle control or 50 ng/mL BTC for an additional 6 h. (**A**) The PTX3 mRNA levels were examined using RT-qPCR (*n* = 4). (**B**) The PTX3 accumulation levels in the conditioned medium were measured using an enzyme immunoassay (*n* = 5). (**C**) SVOG cells were pretreated with either DMSO or the pan-PKC inhibitor, sotrastaurin (10 µM) for 1 h before the treatment with either vehicle control or 50 ng/mL BTC for an additional 6 h. The PTX3 mRNA levels were examined using RT-qPCR (*n* = 4). (**D**, **E**) Primary hGL cells were pretreated with either DMSO or the MEK inhibitor, U0126 (10 µM) for 1 h before the treatment with either vehicle control or 50 ng/mL BTC for an additional 6 h. (**D**) The PTX3 mRNA levels were examined using RT-qPCR (*n* = 4). (**E**) The PTX3 accumulation levels in the conditioned medium were measured using an enzyme immunoassay (*n* = 5). (**F**) primary hGL cells were pretreated with either DMSO or the pan-PKC inhibitor, sotrastaurin (10 µM) for 1 h before the treatment with either a vehicle control or 50 ng/mL BTC for an additional 6 h. The PTX3 mRNA levels were examined using RT-qPCR (*n* = 4). The data presented are the mean ± SEM of at least three independent experiments. Values labeled with different letters are significantly different (*P* < 0.05). BTC, betacellulin; Ctrl, control

In this part of the study, we aimed to examine whether the MEK/ERK signaling pathway is involved in the upregulation of PTX3 induced by EGF-like factors, specifically BTC, in hGL cells. To investigate this, SVOG cells and primary hGL cells were preincubated with U0126, a MEK inhibitor, before the treatment with BTC. The results showed that preincubation with U0126 significantly inhibited the BTC-induced upregulation of PTX3 mRNA levels in both SVOG cells (Fig. 6A) and primary hGL cells (Fig. 6D). Moreover, preincubation with U0126 also suppressed the increase in PTX3 mRNA levels induced by AREG and EREG treatment (Supplemental Fig. 6A-B). Consistent with these findings, the upregulation of PTX3 protein production induced by BTC in the conditioned medium was also blocked by pretreatment with U0126 in both SVOG cells (Fig. 6B) and primary hGL cells (Fig. 6E).

In a previous study, we had shown that BTC could upregulate the phosphorylation level of Connexin 43 in hGL cells via the induction of the protein kinase C (PKC) signaling pathway. To explore whether PKC is involved in the upregulation of PTX3 induced by BTC, we preincubated hGL cells with 10 µM sotrastaurin, a pan-PKC inhibitor, for 1 h before adding BTC. However, the mRNA expression results indicated that the PKC inhibitor had little influence on the BTC-induced increase in PTX3 mRNA expression in both SVOG cells (Fig. 6C) and primary hGL cells (Fig. 6F). This suggests that the PKC signaling pathway is not involved in the BTC-upregulated PTX3 expression in hGL cells. Based on these findings, we concluded that the MEK/ERK signaling pathway mediates the enhancing effects of EGF-like factors, including BTC, AREG, and EREG, on PTX3 expression in hGL cells. This pathway appears to play a crucial role in the regulation of PTX3 expression in response to EGF-like growth factors in these cells.

## Discussion

PTX3 is a critical protein involved in the ovulatory process and is essential for maintaining the stability of the ECM during cumulus expansion [4, 19]. Based on our previous studies, PTX3 was identified as a downstream target of the GC-derived TGF-β superfamily members. Several factors such as growth and differentiation factor 8, TGF-β1, activin A, and various bone morphogenetic proteins (BMPs) have been reported to suppress the expression of PTX3 in human GCs [18–23]. This led us to propose that human GCs may secrete other factors that regulate cumulus expansion induced by the LH surge before the time of ovulation. Additionally, studies have demonstrated that the EGFR-mediated signaling of EGF family members induced by LH surge promote COC expansion and oocyte maturation in preovulatory follicle [24, 25]. ERK1/2, a downstream target of the EGFR pathway in GCs, is essential for regulating genes involved in hyaluronan synthesis and accumulation, which are critical for cumulus expansion [14, 26]. Studies have suggested that AREG-induced activation of calpain 2 and ERK1/2 contributes to COC expansion through cumulus cell detachment and formation of cell surface protrusions. Studies have suggested that AREG-induced activation of Ca^2+^ and ERK1/2 contributes to COC expansion through cumulus cell detachment and formation of cell surface protrusions [27]. Additionally, AREG, BTC, and EREG have been shown to increase the expression of genes related to hyaluronan production and stabilization, promoting cumulus expansion [12]. Furthermore, supplementation of EGF-like factors during in vitro maturation (IVM) has been shown to improve oocyte developmental competence compared to FSH [28] or LH [29]. Understanding the molecular mechanisms underlying EGF-like factors’ cellular activities is essential for developing therapeutic strategies for ovarian disorders and infertility.

In this study, we investigated the role of AREG, BTC, and EREG in regulating PTX3 expression in hGL cells using SVOG cells as a model and employing a siRNA-based knockdown approach. We demonstrated that these EGF-like factors upregulate PTX3 expression by activating the ERK1/2 signaling pathway, which is dependent on EGFR. Our recent study also highlighted the involvement of PKC signaling in BTC-mediated phosphorylation of connexin 43 in hGL cells [11]. However, in this context, our results suggest that PKC is not involved in the regulation of PTX3 induced by EGF-like factors in hGL cells.

Although our in vitro findings provide valuable insights into EGF-like factors’ role in PTX3 expression and COC expansion, there are limitations to consider. The in vitro cell culture systems, such as immortalized hGL cell lines and primary hGL cells from induced ovulation, may not fully replicate the in vivo microenvironment and may have differing receptor expression and cell activities compared to GCs in growing follicles. Our study was conducted using luteal cells that had already been exposed to hCG. This preconditioning of the cells is an essential aspect of our experimental model, as it mimics physiological conditions in vivo, where luteal cells have undergone hCG stimulation. Therefore, further elaboration on the implications of our findings in the context of culture systems and treatments would enhance the comprehensiveness of our manuscript. Additionally, hGL cells are primary cells that are directly isolated from follicular fluid obtained from patients undergoing IVF treatment and have a finite lifespan in culture. They retain many of the characteristics and functions of the ovarian tissue from which they were derived. However, they can only be cultured for a limited number of passages before they stop dividing and eventually senesce. On the other hand, SVOG cells are immortalized cell lines that are derived from primary cells but have undergone genetic modifications to bypass senescence and have an indefinite lifespan in culture. These cell lines can be cultured indefinitely and are often used as convenient models for studying specific cellular processes. However, they may exhibit alterations in gene expression or behavior compared to primary cells, and thus may not fully represent the physiological characteristics of the original tissue. Moreover, further in vivo studies using animal models or clinical samples will be necessary to validate and extend these findings to physiological situations.

**Fig. 7:**
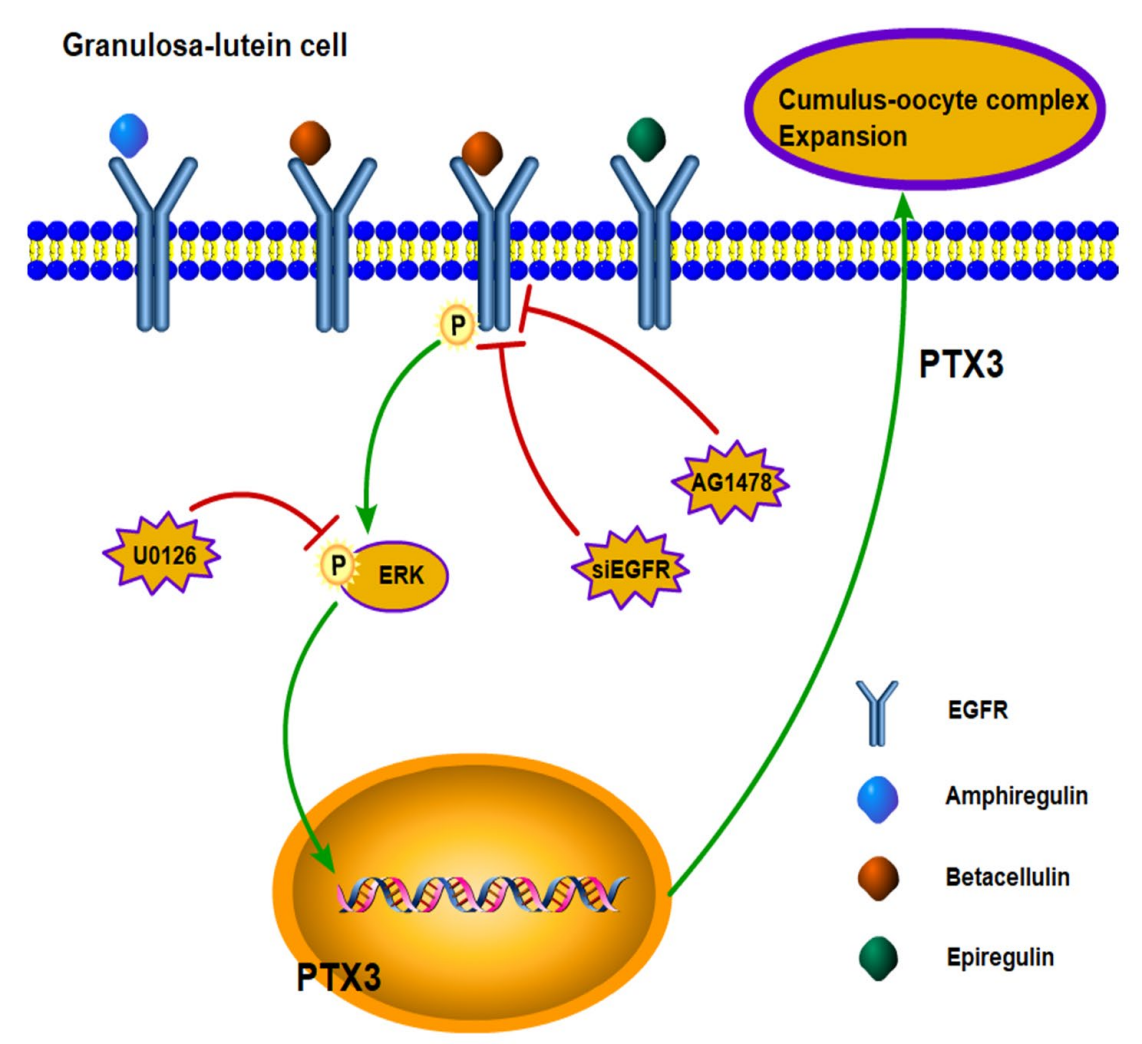
Proposed model for the cell activity of EGF-like factors on the expression of PTX3 in hGL cells. Treatment with EGF-like factors leads to the activation of EGFR, which subsequently induces a signal that activates ERK. The phosphorylated ERK is then translocated into the nucleus where it binds to the PTX3 promoter and increases the transcription of PTX3 in both primary and immortalized hGL cells. AREG, amphiregulin; BTC, betacellulin; EREG, epiregulin; EFGR, Epidermal growth factor receptor; ERK, extracellular signal-regulated kinase

In conclusion, our study demonstrates that AREG, BTC, and EREG upregulate PTX3 expression in hGL cells through the ERK1/2 signaling pathway (Fig. 7). This provides new insights into the molecular mechanisms by which EGF-like factors modulate human COC expansion during the periovulatory stage. Further investigations using animal models and clinical samples will be crucial in understanding the biological significance and potential therapeutic applications of EGF-like factors in regulating PTX3 expression and COC expansion in preovulatory ovaries.

### Electronic supplementary material

Below is the link to the electronic supplementary material.

Supplementary Material 1

## Data Availability

All data generated or analyzed during this study are included in this published article

## Abbreviations

ANOVA: one-way analysis of variance
AREG: amphiregulin
BMP: bone morphogenetic protein
BTC: betacellulin
ECM: extracellular matrix
COC: cumulus-oocyte-complex
EGF: epidermal growth factor
EGFR: epidermal growth factor receptor
EPGN: epigen
ELISA: enzyme-linked immunosorbent assay
EREG: epiregulin
ERK1/2: extracellular signal-regulated kinase 1 and 2
GAPDH: glyceraldehyde-3-phosphate dehydrogenase
GCs: granulosa cells
GJIC: gap junctional intercellular communication
HA: hyaluronan
HB-EGF: heparin-binding EGF-like growth factor
hGL: human granulosa-lutein
IαI: inter-α-trypsin inhibitor
in IVM: vitro maturation
IVF: in vitro fertilization
JAK/STAT: Janus kinase/signal transducers and activators of transcription
MAPK: mitogen-activated protein kinase
PBS: phosphate buffered saline
PTX3: pentraxin 3
NRG1-4: neuregulin 1–4
PI3K: phosphatidylinositol-3 kinase
PKC: protein kinase C
PVDF: polyvinylidene fluoride
RT-qPCR: Reverse transcription quantitative real-time polymerase chain reaction
siRNA: small interfering RNA
RTK: receptor tyrosine kinase
SMAD: Sma- and Mad-related protein
TBS: tris-buffered saline
TGFα: transforming growth factor-α
TGF-β: transforming growth factor-β
TSG-6: tumor necrosis factor-stimulated gene-6

## References

[1] Salustri A, Garlanda C, Hirsch E, De Acetis M, Maccagno A, Bottazzi B (2004). PTX3 plays a key role in the organization of the cumulus oophorus extracellular matrix and in in vivo fertilization. Development.

[2] Zhuo L, Hascall VC, Kimata K (2004). Inter-alpha-trypsin inhibitor, a covalent protein-glycosaminoglycan-protein complex. J Biol Chem.

[3] Fulop C, Szanto S, Mukhopadhyay D, Bardos T, Kamath RV, Rugg MS (2003). Impaired cumulus mucification and female sterility in tumor necrosis factor-induced protein-6 deficient mice. Development.

[4] Varani S, Elvin JA, Yan C, DeMayo J, DeMayo FJ, Horton HF (2002). Knockout of pentraxin 3, a downstream target of growth differentiation factor-9, causes female subfertility. Mol Endocrinol.

[5] Inforzato A, Rivieccio V, Morreale AP, Bastone A, Salustri A, Scarchilli L (2008). Structural characterization of PTX3 disulfide bond network and its multimeric status in cumulus matrix organization. J Biol Chem.

[6] Huang X, Hao C, Shen X, Zhang Y, Liu X (2013). RUNX2, GPX3 and PTX3 gene expression profiling in cumulus cells are reflective oocyte/embryo competence and potentially reliable predictors of embryo developmental competence in PCOS patients. Reprod Biol Endocrinol.

[7] Scarchilli L, Camaioni A, Bottazzi B, Negri V, Doni A, Deban L (2007). PTX3 interacts with inter-alpha-trypsin inhibitor: implications for hyaluronan organization and cumulus oophorus expansion. J Biol Chem.

[8] Schneider MR, Wolf E (2009). The epidermal growth factor receptor ligands at a glance. J Cell Physiol.

[9] Conti M, Hsieh M, Park JY, Su YQ (2006). Role of the epidermal growth factor network in ovarian follicles. Mol Endocrinol.

[10] Park JY, Su YQ, Ariga M, Law E, Jin SL, Conti M (2004). EGF-like growth factors as mediators of LH action in the ovulatory follicle. Science.

[11] Li Y, Chang HM, Sung YW, Zhu H, Leung PCK, Sun YP (2023). Betacellulin regulates gap junction intercellular communication by inducing the phosphorylation of connexin 43 in human granulosa-lutein cells. J Ovarian Res.

[12] Ashkenazi H, Cao X, Motola S, Popliker M, Conti M, Tsafriri A (2005). Epidermal growth factor family members: endogenous mediators of the ovulatory response. Endocrinology.

[13] Herbst RS (2004). Review of epidermal growth factor receptor biology. Int J Radiat Oncol Biol Phys.

[14] Hsieh M, Lee D, Panigone S, Horner K, Chen R, Theologis A (2007). Luteinizing hormone-dependent activation of the epidermal growth factor network is essential for ovulation. Mol Cell Biol.

[15] Shimada M, Hernandez-Gonzalez I, Gonzalez-Robayna I, Richards JS (2006). Paracrine and autocrine regulation of epidermal growth factor-like factors in cumulus oocyte complexes and granulosa cells: key roles for prostaglandin synthase 2 and progesterone receptor. Mol Endocrinol.

[16] Bolamba D, Floyd AA, McGlone JJ, Lee VH (2002). Epidermal growth factor enhances expression of connexin 43 protein in cultured porcine preantral follicles. Biol Reprod.

[17] Gall L, Chene N, Dahirel M, Ruffini S, Boulesteix C (2004). Expression of epidermal growth factor receptor in the goat cumulus-oocyte complex. Mol Reprod Dev.

[18] Chang HM, Cheng JC, Fang L, Qiu X, Klausen C, Taylor EL (2015). Recombinant BMP4 and BMP7 downregulate pentraxin 3 in human granulosa cells. J Clin Endocrinol Metab.

[19] Nagyova E, Kalous J, Nemcova L (2016). Increased expression of pentraxin 3 after in vivo and in vitro stimulation with gonadotropins in porcine oocyte-cumulus complexes and granulosa cells. Domest Anim Endocrinol.

[20] Chang HM, Cheng JC, Leung PC (2013). Theca-derived BMP4 and BMP7 down-regulate connexin43 expression and decrease gap junction intercellular communication activity in immortalized human granulosa cells. J Clin Endocrinol Metab.

[21] Li H, Chang HM, Shi Z, Leung PCK (2018). SNAIL mediates TGF-beta1-Induced downregulation of Pentraxin 3 expression in human granulosa cells. Endocrinology.

[22] Liu C, Chang HM, Yi Y, Fang Y, Zhao F, Leung PCK (2019). ALK4-SMAD2/3-SMAD4 signaling mediates the activin A-induced suppression of PTX3 in human granulosa-lutein cells. Mol Cell Endocrinol.

[23] Bai L, Chang HM, Cheng JC, Chu G, Leung PCK, Yang G (2017). ALK2/ALK3-BMPR2/ACVR2A mediate BMP2-Induced downregulation of Pentraxin 3 expression in human granulosa-lutein cells. Endocrinology.

[24] Downs SM, Chen J (2008). EGF-like peptides mediate FSH-induced maturation of cumulus cell-enclosed mouse oocytes. Mol Reprod Dev.

[25] Reizel Y, Elbaz J, Dekel N (2010). Sustained activity of the EGF receptor is an absolute requisite for LH-induced oocyte maturation and cumulus expansion. Mol Endocrinol.

[26] Panigone S, Hsieh M, Fu M, Persani L, Conti M (2008). Luteinizing hormone signaling in preovulatory follicles involves early activation of the epidermal growth factor receptor pathway. Mol Endocrinol.

[27] Kawashima I, Liu Z, Mullany LK, Mihara T, Richards JS, Shimada M (2012). EGF-like factors induce expansion of the cumulus cell-oocyte complexes by activating calpain-mediated cell movement. Endocrinology.

[28] Richani D, Ritter LJ, Thompson JG, Gilchrist RB (2013). Mode of oocyte maturation affects EGF-like peptide function and oocyte competence. Mol Hum Reprod.

[29] Prochazka R, Petlach M, Nagyova E, Nemcova L (2011). Effect of epidermal growth factor-like peptides on pig cumulus cell expansion, oocyte maturation, and acquisition of developmental competence in vitro: comparison with gonadotropins. Reproduction.

