# Supplementary material for: EGF-like growth factors upregulate pentraxin 3 expression in human granulosa-lutein cells
Source: J Ovarian Res. 2024 May 8;17:97. doi: 10.1186/s13048-024-01404-5 (PMC11077866; doi:10.1186/s13048-024-01404-5)
Supplement: Supplementary file 1 — Supplementary Material 1 [file 13048_2024_1404_MOESM1_ESM.docx]

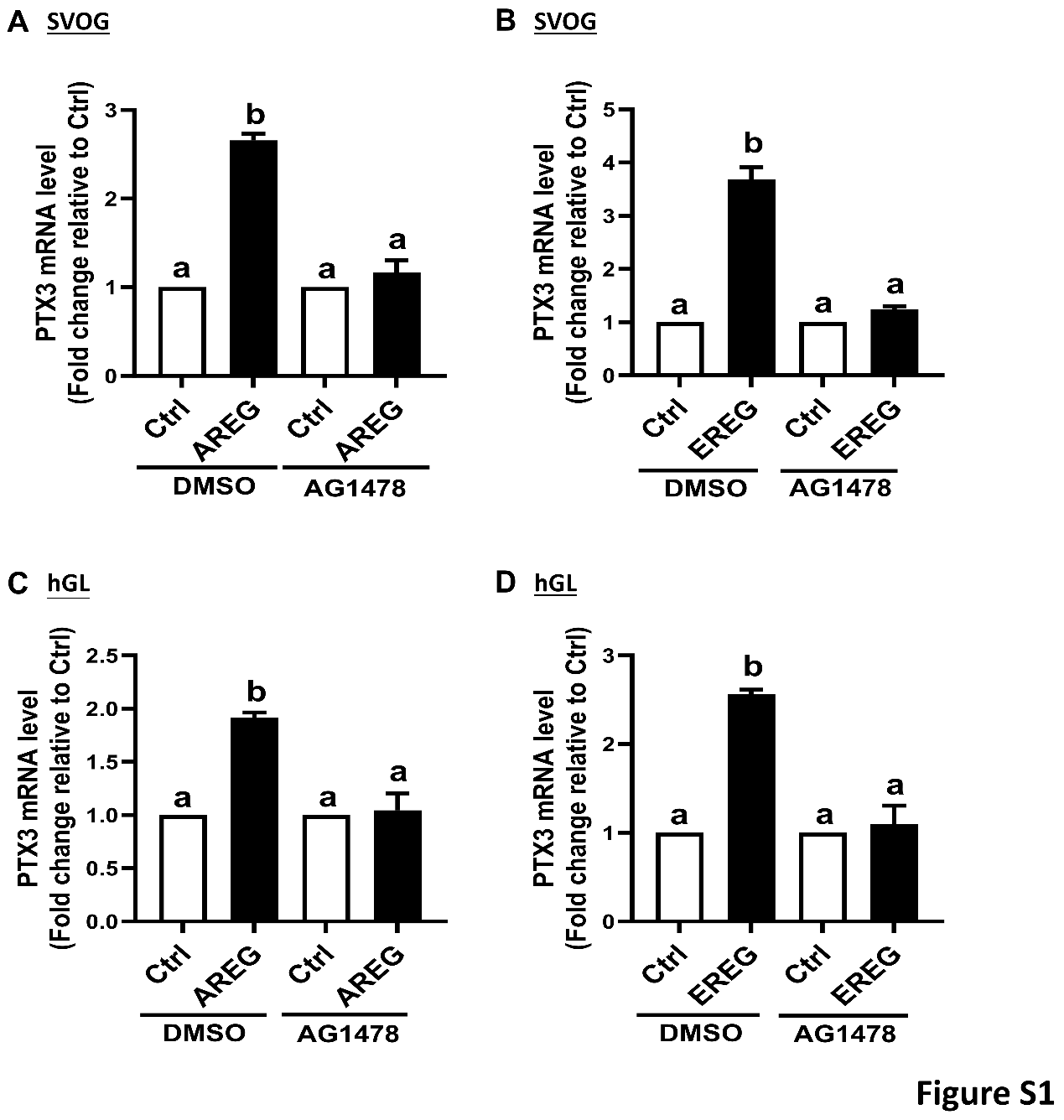


**Supplemental Figure 1. EGFR mediates the upregulation of PTX3 induced by AREG or EREG in hGL cells.** (A, B) SVOG cells were pretreated with either dimethyl sulfoxide (DMSO) or the specific EGFR inhibitor, AG1478 (10 μM) for 1 h and then treated with either vehicle control or 50 ng/mL of AREG (A) or EREG (B) for an additional 6 h. The PTX3 mRNA levels were examined using RT-qPCR (n=4). (C, D) Primary hGL cells were pretreated with either DMSO or the specific EGFR inhibitor, AG1478 (10 μM) for 1 h and then treated with either vehicle control or 50 ng/mL of AREG (C) or EREG (D) for an additional 6 h. The PTX3 mRNA levels were examined using RT-qPCR (n=4). The data presented are the mean ± SEM of at least three independent experiments. Values labeled with different letters are significantly different (P< 0.05). AREG, amphiregulin; EREG, epiregulin; Ctrl, control.





**Supplemental Figure 2. EGFR mediates the upregulation of PTX3 induced by AREG or EREG in SVOG cells.** SVOG cells were transfected with either siCtrl or siEGFR for 48 h and then treated with either vehicle control or 50 ng/mL of AREG (A) or EREG (B) for an additional 6 h. The PTX3 mRNA levels were examined using RT-qPCR (n=3). (C, D) The knockdown efficiency and specificity of siEGFR were examined using RT-qPCR (n=4). The results are expressed as the mean ± SEM of at least three independent experiments. Values marked with different letters are significantly different (P< 0.05). AREG, amphiregulin; EREG, epiregulin; Ctrl, control.





**Supplemental Figure 3. EGFR mediates the upregulation of PTX3 induced by AREG or EREG in primary hGL cells.** Primary hGL cells were transfected with either siCtrl or siEGFR for 48 h and then treated with either vehicle control or 50 ng/mL of AREG (A) or EREG (B) for an additional 6 h. The PTX3 mRNA levels were examined using RT-qPCR (n=5). (C, D) The knockdown efficiency and specificity of siEGFR were examined using RT-qPCR (n=5). The data presented are the mean ± SEM of at least three independent experiments. Values labeled with different letters are significantly different (P< 0.05). AREG, amphiregulin; EREG, epiregulin; Ctrl, control.


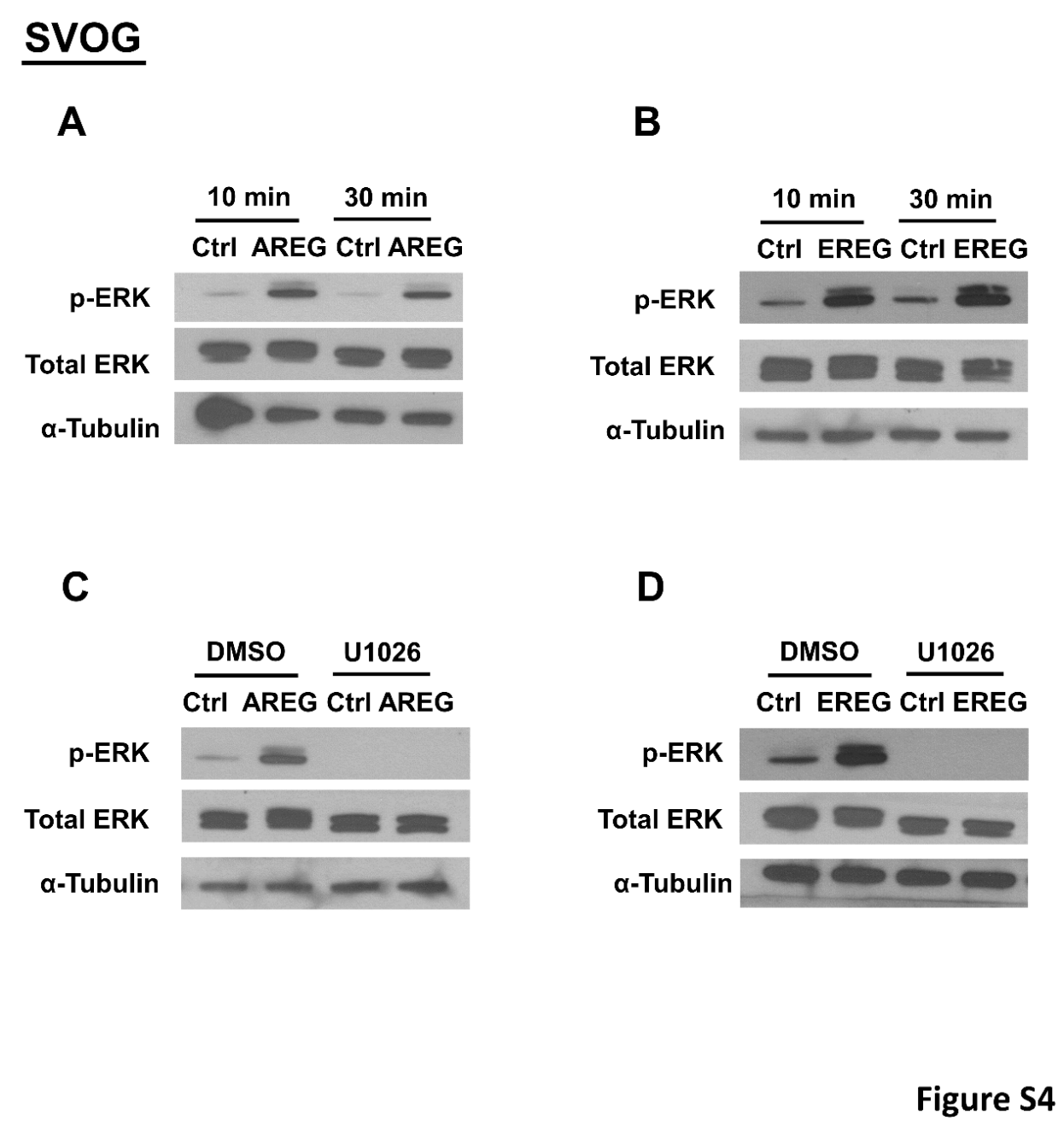


**Supplemental Figure 4. AREG or EREG activates MEK/ERK signaling pathway in SVOG cells.** (A, B) SVOG cells were treated with either vehicle control or 50 ng/mL AREG (A) or EREG (B) for 10 min or 30 min (n=4). (C, D) SVOG cells were pretreated with either DMSO or the MEK inhibitor, U0126 (10 μM) for 1 h before the treatment with either vehicle control or 50 ng/mL AREG (C) or EREG (D) for an additional 30 min (n=4). The phosphorylated protein levels of ERK1/2 were examined using Western blot analysis. The data presented are the mean ± SEM of at least three independent experiments. Values labeled with different letters are significantly different (P< 0.05). AREG, amphiregulin; EREG, epiregulin; Ctrl, control.


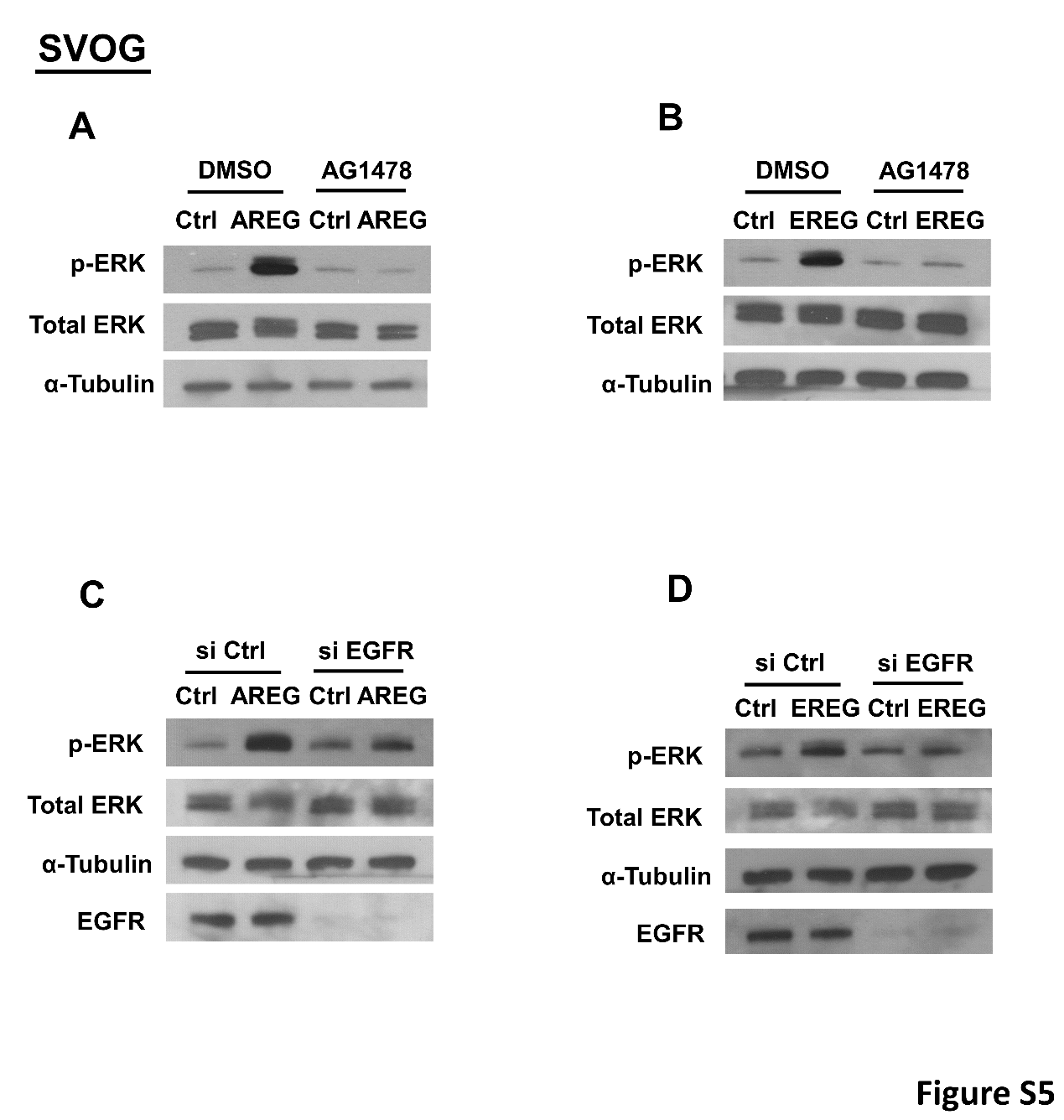


**Supplemental Figure 5. EGFR is needed for the activation of the MEK/ERK signaling pathway induced by AREG or EREG in SVOG cells.** (A, B) SVOG cells were pretreated with either DMSO or the EGFR inhibitor, AG1478 (10 μM) for 1 h before the treatment with either vehicle control or 50 ng/mL AREG (A) or EREG (B) for an additional 30 min (n=4). (C, D) SVOG cells were transfected with either siCtrl or siEGFR for 48 h before the treatment with either vehicle control or 50 ng/mL AREG (C) or EREG (D) for an additional 30 min. The phosphorylated protein levels of ERK1/2 were examined using Western blot analysis (n=4). The data presented are the mean ± SEM of at least three independent experiments. Values labeled with different letters are significantly different (P< 0.05). AREG, amphiregulin; EREG, epiregulin; Ctrl, control.





**Supplemental Figure 6. The activation of MEK/ERK signaling pathway induced by AREG or EREG mediates the upregulation of PTX3 in SVOG cells.**

SVOG cells were pretreated with either DMSO or the MEK inhibitor, U0126 (10 μM) for 1 h before the treatment with either vehicle control or 50 ng/mL AREG (A) or EREG (B) for an additional 6 h. The PTX3 mRNA levels were examined using RT-qPCR (n=3). The data presented are the mean ± SEM of at least three independent experiments. Values labeled with different letters are significantly different (P< 0.05). AREG, amphiregulin; EREG, epiregulin; Ctrl, control.
